# RETRACTION: The Efficacy and Safety of NeubotulinumtoxinA for the Treatment of Forehead Horizontal Lines in Asians – A Clinical, Prospective, Interventional, Split‐Face Study

**DOI:** 10.1111/srt.70287

**Published:** 2025-10-29

**RETRACTION**: J. J. Sy, R. Wu, J. Wan, S.‐B. Kim, and K.‐H. Yi, “The Efficacy and Safety of NeubotulinumtoxinA for the Treatment of Forehead Horizontal Lines in Asians – A Clinical, Prospective, Interventional, Split‐Face Study,” *Skin Research and Technology *30, no. 4 (2024): e13644, https://doi.org/10.1111/srt.13644.

The above article, published online on 27 March 2024 in Wiley Online Library (wileyonlinelibrary.com), has been retracted by agreement between *Skin Research and Technology*; and John Wiley & Sons Ltd. The article was accepted for publication following an insufficient peer review process that does not meet the standards of the journal's peer review policy. Furthermore, scientific inconsistencies between data presented in figures and results discussed in the text were identified, and the article lacks essential data and information to interpret and reproduce the findings. Accordingly, the article has been retracted. The authors have been informed of the retraction decision but were unavailable for final confirmation.

